# Targeting the Shift from M1 to M2 Macrophages in Experimental Autoimmune Encephalomyelitis Mice Treated with Fasudil

**DOI:** 10.1371/journal.pone.0054841

**Published:** 2013-02-13

**Authors:** Chunyun Liu, Yanhua Li, Jiezhong Yu, Ling Feng, Shaowei Hou, Yueting Liu, Mingfang Guo, Yong Xie, Jian Meng, Haifei Zhang, Baoguo Xiao, Cungen Ma

**Affiliations:** 1 Institute of Brain Science, Department of Neurology, Medical School, Shanxi Datong University, Datong, China; 2 Institute of Neurology, Huashan Hospital, Institutes of Brain Science and State Key Laboratory of Medical Neurobiology, Fudan University, Shanghai, China; 3 Department of Neurology, Shanxi University of Traditional Chinese Medicine, Taiyuan, China; University of Lyon, France

## Abstract

We observed the therapeutic effect of Fasudil and explored its mechanisms in experimental autoimmune encephalomyelitis (EAE), an animal model of multiple sclerosis (MS). Fasudil, a selective Rho kinase (ROCK) inhibitor, was injected intraperitoneally at 40 mg/kg/d in early and late stages of EAE induction. Fasudil ameliorated the clinical severity of EAE at different stages, and decreased the expression of ROCK-II in spleen, accompanied by an improvement in demyelination and inhibition of inflammatory cells. Fasudil mainly inhibited CD4^+^IL-17^+^ T cells in early treatment, but also elevated CD4^+^IL-10^+^ regulatory T cells and IL-10 production in late treatment. The treatment of Fasudil shifted inflammatory M1 to anti-inflammatory M2 macrophages in both early and late treatment, being shown by inhibiting CD16/32, iNOS, IL-12, TLR4 and CD40 and increasing CD206, Arg-1, IL-10 and CD14 in spleen. By using Western blot and immunohistochemistry, iNOS and Arg-1, as two most specific markers for M1 and M2, was inhibited or induced in splenic macrophages and spinal cords of EAE mice treated with Fasudil. In vitro experiments also indicate that Fasudil shifts M1 to M2 phenotype, which does not require the participation or auxiliary of other cells. The polarization of M2 macrophages was associated with the decrease of inflammatory cytokine IL-1β, TNF-α and MCP-1. These results demonstrate that Fasudil has therapeutic potential in EAE possibly through inducing the polarization of M2 macrophages and inhibiting inflammatory responses.

## Introduction

Multiple sclerosis (MS) is an immune-mediated chronic inflammatory demyelinating disease of the central nervous system (CNS), destroying the myelin and the axon in variable degrees. The etiology of MS is still not known, a combination of several factors may be involved, including genetics, environment, and possibly a virus [1]. But, the weight of evidence would favour a significantly greater role for the environment over genetics [2]. During the development of MS, autoreactive T cells and macrophages which are stimulated in peripheral lymphoid tissues, infiltrate into the CNS and produce inflammatory molecules, leading to oligodendrocyte death and axonal damage in the CNS [3], [4]. Thus, the pathogenesis of MS may be related to activation, migration and effector function of immune cells as well as their products such as cytokines, chemokines, adhesion molecules or other inflammatory factors [5]–[7]. These constitute the modern immunological basis for the development of novel clinical and preclinical immunomodulatory therapies for MS [8].

Experimental autoimmune encephalomyelitis (EAE), a well-established animal model of MS, is characterized by inflammation and demyelination of the CNS [9], which exhibits some of the symptomatology and pathology observed in MS patients. The immunopathogenesis of EAE involves the disruption of the blood brain barrier (BBB) [10] and the integrated attack of T cells, macrophages, and dendritic cells [11]. However, the dynamic balance between the Th1/Th17 and Treg cells controls the development of EAE [12]. The pathogenesis of EAE can be controlled by T cells and/or macrophages with suppressor function [13]–[15]. CD4^+^CD25^+^ regulatory T cells (Treg) purified from adoptive EAE mice confer protection against active EAE by producing IL-10 [16]. Various subsets of Treg cells including natural Tregs (nTregs), IL-10 producing type 1 Tregs (Tr1 cells), transforming growth factor-β producing Th3 cells, CD8^+^ Tregs, and CD4^+^CD25^+^ Tregs in MS and EAE also control the initiation and progression of disease and even treat it [17].

Macrophages are also the major effector cells mediating immune responses in EAE. They act as antigen presenting cells (APC), thereby activating an antigen-specific T cell response in the periphery and the CNS. Recent studies have shown that macrophages are an extremely heterogeneous lineage, displaying a combination of inflammatory and anti-inflammatory functions [18]. The two extremes in the spectrum of macrophage function are represented by the classically activated (or M1) and the alternative activated (or M2) phenotypes [19]. Classically activated macrophages, which are induced by a combination of IFN-γ and LPS/ TNF-α, have anti-microbial and cytotoxic properties, whereas alternatively activated macrophages are anti-inflammatory or reparative [20], [21].

Rho-kinase (ROCK), a serine/threonine kinase of molecular mass 160 kDa, is activated by binding to the active GTP-bound form of the small GTPase Rho, and expressed both centrally and peripherally, which contributes to fundamental cellular processes including migration, proliferation and survival [22], [23]. A series of studies have demonstrated that ROCK inhibitor Y-27632 reduced leukocyte infiltration in several models of inflammation such as ischemic heart injury [24], endotoxic liver [25], and lung injury [26]. Fasudil (1-(5-isoquinolinesulfonyl)-homo-piperazine), a selective ROCK inhibitor, has been used to treat cerebral vasospasm [27], stroke [28] and traumatic spinal cord injury [29]. In addition, the inhibition of ROCK enhanced oligodendrocyte precursor maturation into the oligodendrocyte lineage [30]. Therefore, the blockade of the Rho/ROCK system is considered to be beneficial for CNS inﬂammatory demyelination. Thus, targeting ROCK inhibition is a promising strategy to achieve neuroprotection, axonal regeneration, oligodendrocyte generation and inflammation suppression. In the present study, we observed therapeutic potential of Fasudil in different phases of EAE induction, and explored novel mechanisms of action.

## Materials and Methods

### Animals

Female C57BL/6 mice (8–10 weeks and 18–20 g) were purchased from Vital River Laboratory Animal Technology Co. Ltd. (Beijing, China). All experiments were conducted in accordance with the guidelines of the International Council for Laboratory Animal Science. The study was approved by the Ethics Committee of Shanxi Datong University, Datong, China. All mice were housed under pathogen-free conditions and maintained in a reversed 12∶12-hours light/dark cycle in a temperature-controlled room (25 ± 2°C) for one week prior to experimental manipulation.

### Induction and Clinical Evaluation of EAE

Mouse myelin oligodendrocyte glycoprotein peptide_35–55_ (MOG_35–55,_ MEVGWYRSPFSRVVHLYRNGK) was produced in an automatic synthesizer (CL. Bio-Scientific. Company, Xian, China). The purity of the peptide was >95% as determined by HPLC.

Chronic EAE was induced by subcutaneous immunization on the upper dorsal flanks with 300 µg of MOG_35–55_ in Freund’s complete adjuvant (Sigma, USA) supplemented with 3 mg/ml of *M. Tuberculosis H37Ra* (BD Difco, USA) (400 µg/mice). Mice were injected with 400 ng of pertussis toxin (Enzo Life Sciences, USA) via abdominal cavity at the same time of immunization and again 48 hour later. Animals were weighed and evaluated for clinical score every other day in a blinded fashion by at least two investigators. Once clinical score of EAE was 3, we gave special care to the mice, which is necessary to soften the food with water in dish, add nutrients such as egg, and put dish in the bottom of the cage, making it easy to get food, water and nutrition. Clinical score of EAE was graded according to the following criteria: 0. healthy; 1. limp tail; 2. ataxia and/or paresis of hindlimbs; 3. paralysis of hindlimbs and/or paresis of forelimbs; 4. tetraparalysis; 5. moribund or death. All animal experiments were repeated three times.

### Administration of Fasudil

The animals were divided into 3 groups. Fasudil early treatment (n = 28): Fasudil (Tianjin Chase Sun Pharmaceutical Co., Ltd) dissolved in normal saline (NS) was injected intraperitoneally (i.p.) at 40 mg/kg/d every other day on day 3 post-immunization (p.i.) till day 27 p.i. Fasudil late treatment (n = 28): all mice this group were injected with Fasudil in a similar manner, when a mouse showed clinical symptoms. EAE control (n = 31): The injection of NS was set up in a similar manner. The dose of Fasudil in this study was selected according to preliminary experiments and clinical dosage. Thirteen mice were sacrificed on day 28 p.i. for clinical evaluation, while other mice were respectively sacrificed on days 9 and 21 p.i. for laboratory examination.

### Histology and Immunohistochemistry

On day 21 p.i., mice were perfused with saline and 4% buffered paraformaldehyde. The spleens and spinal cords (lower thoracic-lumbar) were sliced (10 µm) and the pathological changes were detected by hematoxylin and eosin (H&E) staining and Luxol Fast Blue staining. For immunohistochemistry, non-specific binding was blocked with 3% bovine serum (Serotec, UK), and permeabilized with 0.3% Triton X-100 in 1% BSA-PBS for 30 min. The sections were incubated at 4°C overnight with anti-ROCK-II (1∶1000; clone A60, Sigma, USA), anti-CD68 (Serotec, UK), anti-CD11b (eBiosence Inc, USA), anti-iNOS (1∶200, Cayman Chemicals Company, USA) and anti-Arg-1 (1∶300, Cayman Chemicals Company), then incubated with corresponding secondary antibodies at room temperature (RT) for 2 h. As a negative control, additional sections were treated similarly, but the primary antibodies were omitted. For each animal, four sections in each sample were examined in a blinded fashion.

### Preparation of Mononuclear Cells and Macrophages

On days 9 and 21 p.i., mice were sacrificed and spleens were removed under aseptic conditions. Suspensions of mononuclear cells (MNCs) from spleens were prepared by grinding the organ through a 40 µm nylon mesh in medium. Erythrocytes in the suspensions were osmotically lysed. Cells were then washed 3 times and re-suspended in medium. Cells were adjusted to 3×10^6^/ml.

For the preparation of macrophages, splenic MNC were seeded in 6-well tissue culture plates (Corning, 2×10^6^ cells/ml) and allowed to adhere for 1 h at 37°C in a humidified atmosphere containing 5% CO_2_. Non-adherent cells were removed, and adherent cells were further purified by positive selection using CD11b microbeads (Miltenyi Biotec, Leiden, The Netherlands). The purity of CD11b^+^ cells was evaluated by flow cytometry (CD11b^+^F4/80^+^ cells >96%). In addition, purified macrophages from healthy mice were isolated and used for in vitro experiment.

### Treatment of IFN-γ and Fasudil in vitro

Preliminary experiments were carried out to determine the optimal concentration and exposure time of IFN-γ in purified macrophages (data not shown). In the in vitro experiment, macrophages were stimulated in medium containing 100 U/ml of IFN-γ (Sigma, Louis, MO). To study the effect of Fasudil in IFN-γ-activated macrophages, Fasudil (15 µg/ml) was added for 12 h immediately after IFN-γ treatment.

### Flow Cytometry Analysis

On days 9 and 21 p.i., mice were sacrificed and spleens were removed under aseptic conditions. For cell surface staining, MNCs were stained for 20 min at RT in 1% BSA-PBS buffer with the following panel of antibodies: FITC-CD4 (eBioscience, USA) and PE-CD25 (eBioscience), Alexa Fluor 488-anti-F4/80 (Serotec, UK) and PE-CD16/32, PE-CD206, PE-TLR4, PE-CD11c, PE-CD14, PE-CD40, PE-CD23 and PE-CD200 (eBioscience).

For intracellular staining, MNCs were stimulated with 1 µg/ml brefeldin A (Sigma, USA) for 6 h at 37°C under a 5% CO_2_/95% air atmosphere, and stained for 20 min at RT in 0.3% saponin/1% BSA-PBS buffer with the following panel of antibodies: FITC-CD4 and PE-IL-10, PE-IL-12, PE-IL-17 (eBioscience Inc), and Alexa Fluor 488-anti-F4/80, PE-IL-10, PE-IL-12, anti-iNOS and anti-arginase-1 (Arg-1), followed by corresponding PE-conjugated secondary antibodies for iNOS and Arg-1. Cells were gated using forward and sideward scatter characteristics for lymphocytes and monocytes and at least 10,000 gated events were collected using flow cytometer (BD Biosciences, USA). Data were analyzed using CellQuest software.

### Cytokine ELISA Assay

On day 21 p.i., mice were sacrificed and spleens were removed under aseptic conditions. Splenic MNCs (6×10^5^/200 µl/well) were incubated for 48 h at 37°C in the presence of MOG_35–55_ (10 µg/ml). Supernatants were harvested and measured for cytokine concentrations of IL-1β (Invitrogen Inc, USA), IL-6, TNF-α, MCP-1 (Pepro tech Inc, USA), IL-10 and IL-17 (eBioscience Inc) by a sandwich ELISA kits following the manufacturer’s instructions. Determinations were performed in duplicate in 3 independent experiments. The results were expressed as pg/ml.

### Western Blot Analysis

On day 21 p.i., mice were perfused with saline, and spleens and spinal cords were homogenized with a microcontent motor-operated tissue homogenizer (Kimble Kontes, USA), using protein extraction kit (Millipore) supplemented with a cocktail of protease inhibitors. The homogenates were centrifuged at 20,000 g for 20 min at 4°C, the supernatants were collected. The protein from macrophages was also isolated in a similar manner.

Protein concentrations were determined by a Bradford protein assay. Equal amounts of protein (30 µg) were separated by SDS-PAGE and electroblotted onto nitrocellulose membrane (Immobilon-P, Millipore). After non-specific binding was blocked with 5% non-fat dry milk, membranes were incubated at 4°C overnight with anti-iNOS (1∶200, Cayman Chemicals Company, USA), anti-Arginase-1 (Arg-1) (1∶300, Cayman Chemicals Company) and anti-GAPDH (1∶1,000, Epitomics, USA). Bands were visualized with an enhanced chemiluminescence (ECL) system (GE Healthcare Life Sciences, USA). To compare protein loading, antibody directed against GAPDH was used.

### Statistical Analysis

All the experiments were repeated two or three times and GraphPad Prism software was used for statistical analysis. For clinical mean score, nonparametric Kruskal-Wallis test was performed to determine whether an overall statistically significant change existed before Mann Whitney U test to analyze the difference between any two groups. Student’s t-test was used to compare demyelination, inflammation and in vitro experiments. Statistical significance for the other results was analyzed using Two-way ANOVA with multiple comparison post test (Bonferroni). A statistically significant difference was assumed at *p*<0.05.

## Results

### Fasudil Delays Onset, and Ameliorates Severity of EAE

**Figure 1:**
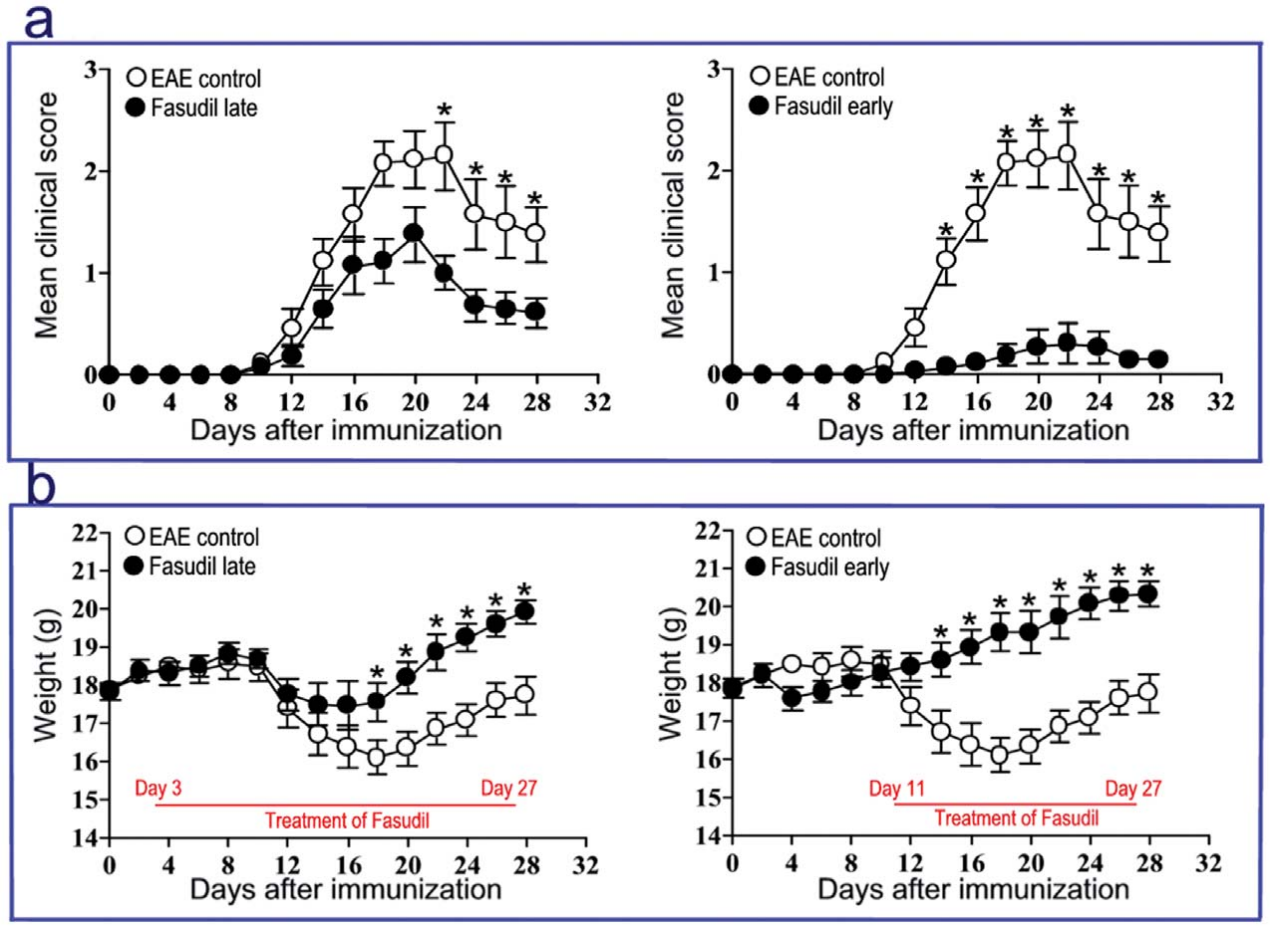
Fasudil delays onset, and ameliorates severity of EAE. Chronic EAE was induced in C57BL/6 mice with MOG_35–55._ Fasudil was injected (i.p.) at 40 mg/kg/d every other day on day 3 p.i. (Fasudil early treatment, n = 13) or on day 11 p.i. (Fasudil late treatment, n = 13). The horizontal line is treatment period of Fasudil. The injection of normal saline was set up as control (EAE control, n = 13) in a similar manner. a) Mean clinical score, b) Mean body weight. The comparison in each time point was separately analyzed by Mann Whitney U test after nonparametric Kruskal-Wallis test. *p<0.05; **p<0.01.

To observe the therapeutic effect of Fasudil at different phases of EAE induction, Fasudil was injected i.p. on days 3 and 11 p.i. As shown in Table 1 and Fig. 1a, the incidence of EAE (23.1%) in Fasudil early-treated mice was decreased as compared with EAE control (100%) and Fasudil late-treated mice (76.9%). In EAE group, mean onset date was 12.38±1.45, mean maximum score was 2.62±1.08. The administration of Fasudil in late-treated mice slightly delayed onset (13.50±2.17, *p*>0.05), and declined maximum clinical score (1.41±1.08, *p*<0.05). The administration of Fasudil in early-treated mice was able to delay clinical onset (18.00±3.61, *p*<0.05), and showed obvious decline in maximum clinical score (0.30±0.72, *p*<0.01). There was a relationship between loss of body weight and severity of clinical score during the course of EAE (Fig. 1b).

**Table 1:** The clinical evaluation of EAE mice.

Group	n	Incidence ( % )	Mean onset date	Mean score of maximal symptom
EAE control	13	100	12.38±1.45	2.62±1.08
Fasudil late	13	76.9	13.50±2.17	1.41±1.08*
Fasudil early	13	23.1*	18.00±3.61*	0.30±0.72**

Fasudil was injected (i.p.) at 40 mg/kg/d every other day on day 3 p.i. (Fasudil early treatment) and on day 11 p.i. (Fasudil late treatment). The injection of normal saline was set up as control (EAE control) in a similar manner. Data are presented as mean ± S.E.M.

* *p*<0.05,

** *p*<0.01.

### Fasudil Improves Demyelination and Inhibits Inflammation in Spinal Cords

**Figure 2:**
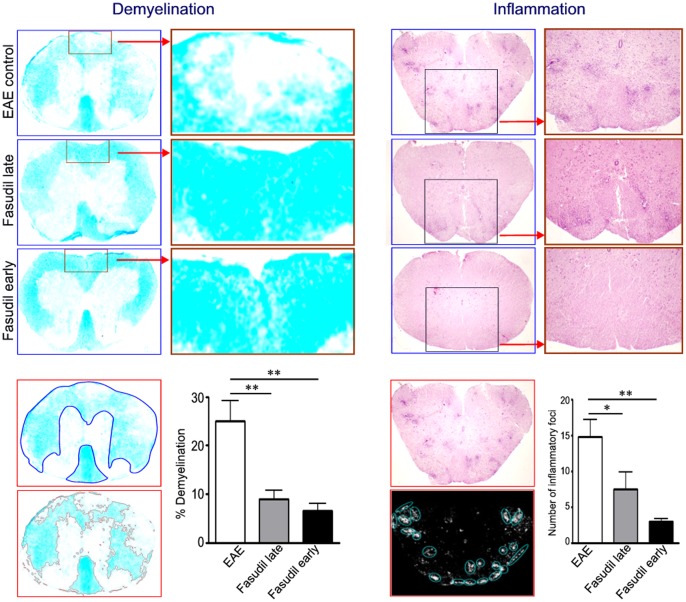
Fasudil inhibits inflammation and improves myelination in spinal cords. Chronic EAE was induced in C57BL/6 mice with MOG_35–55._ Fasudil was injected (i.p.) at 40 mg/kg/d every other day on day 3 p.i. (Fasudil early treatment, n = 9) or on day 11 p.i. (Fasudil late treatment, n = 9). The injection of normal saline was set up as control (EAE control, n = 12) in a similar manner. On day 21 p.i., spinal cords were used for Luxol fast blue and H&E staining. Left) Demyelination stained with Luxol Fast Blue. Total white matter in Luxol Fast Blue was manually outlined, and pixel area (%) of demyelination in total white matter was calculated by Image-Pro Plus software. Right) Inflammation stained with H&E. Color photo in H&E stain was automatically converted to black and white, and number of inflammatory foci (>20 mononuclear cells/focus) in whole spinal cords were calculated by Image-Pro Plus software. Quantitative results are analyzed for 6 mice in each group and are representative of 3 experiments with similar results. *p<0.05, **p<0.01.

We evaluated the pathology of CNS inflammation and demyelination. As shown in Fig. 2, mice with EAE showed extensive myelin loss (demyelination) associated with infiltration of immune cells (inflammation) in spinal cords. When compared with EAE control, Fasudil-treated mice had a significant improvement in the extent of demyelination (*p*<0.01 as compared with late and early treatment, respectively) and inflammation (*p*<0.05 and *p*<0.01 as compared with late and early treatment, respectively) in spinal cords.

### Fasudil Decreases the Expression of ROCK-II in Spleen

**Figure 3:**
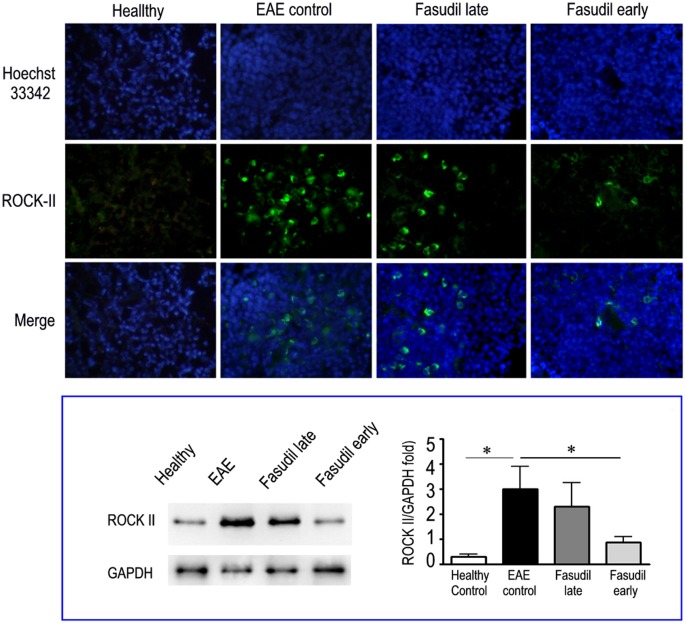
Fasudil inhibits the expression of ROCK-II in spleen. Chronic EAE was induced in C57BL/6 mice with MOG_35–55._ Fasudil was injected (i.p.) at 40 mg/kg/d every other day on day 3 and 11 p.i. On day 21 p.i., spleens were collected, and the expression of ROCK II was measured by immunohistochemistry. Spleen from healthy mice was measured as control. Up) Immunohistochemistry. Green = ROCK-II; Blue = Hoechst nuclear staining. As a negative control, additional section was treated similarly, but the primary antibodies were omitted. Quantitative results are analyzed by Western blot. Down) Western blot. Left down = representative bands from one of three experiments with similar results; Right down = quantitative results are analyzed for 5 mice in each group and expressed as fold change relative to GAPDH as the loading control *p<0.05.

We analyzed the expression of ROCK-II in spleen among three groups. Fig. 3 showed the treatment of Fasudil inhibited the expression of ROCK-II in spleens compared with EAE control, especially in early treatment. We next observed whether we could find the similar phenomenon in Western blot. Indeed, the expression of ROCK-II slightly decreased in Fasudil late-treated mice and significantly declined in Fasudil early-treated mice (*p*<0.05, Fig. 3).

### Fasudil Inhibits IL-17 T Cells in Early and Late Treatment of EAE Induction

**Figure 4:**
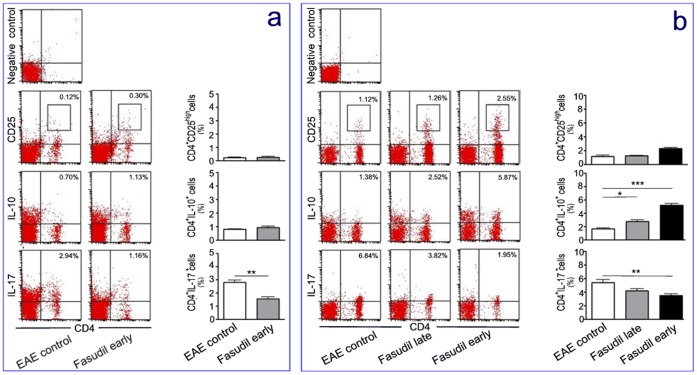
Fasudil induces the regulatory T cells and inhibits IL-17 T cells. Chronic EAE was induced in C57BL/6 mice with MOG_35–55_. Fasudil was injected (i.p.) at 40 mg/kg/d every other day on days 3 and 11 p.i. Mice were sacrificed and splenic MNCs were prepared on days 9 (a) and 21 (b) p.i. Cells were stained with T cell marker CD4 and CD25, IL-10 and IL-17, and subsets of T cells were analyzed using flow cytometry. Scatterplot, representative dot plots from one of three experiments with similar results. Histogram, quantitative results are analyzed for 5 mice in each group. Results are expressed as the percentage of double positive cells in four-quadrant diagram or within rectangle. *p<0.05, **p<0.01, ***p<0.001.

The subsets of CD4^+^CD25^high^, CD4^+^IL-10^+^ and CD4^+^IL-17^+^ T cells in spleen were analyzed on days 9 and 21 p.i. by flow cytometry. On day 9 p.i., percentage of CD4^+^ cells expressing IL-17 in Fasudil early-treated mice was significantly decreased compared with EAE control (p<0.01, Fig. 4a). On day 21 p.i. percentages of CD4^+^ cells expressing IL-10 and IL-17 in EAE control were 1.61±0.26% and 5.36±1.02%, respectively (Fig. 4b). The administration of Fasudil enhanced the population of CD4^+^ cells expressing IL-10 (late treatment = 2.71±0.65% and early treatment = 5.17±0.58%, *p*<0.05 and *p*<0.001 as compared with EAE control, respectively) and decreased the population of CD4^+^ cells expressing IL-17 (3.46±0.77%, *p*<0.01) in early-treated mice (Fig. 4b).

In addition, the proportion of CD4^+^CD25^high^ T regulatory cells in spleen was also determined by flow cytometry. The administration of Fasudil did not influence the population of CD4^+^CD25^high^ cells on day 9 p.i. (Fig. 4a), and slightly elevated percentage of CD4^+^CD25^high^ cells on day 21 p.i. in early-treated mice, but *did not reach statistical significance (* *Fig. 4b* *)*. Therefore, the results suggest that Fasudil inhibited CD4^+^IL-17^+^ T cells in early and late treatment of EAE induction.

### Fasudil Shifts M1 to M2 Phenotype in Early and Late Treatment of EAE Induction

**Figure 5:**
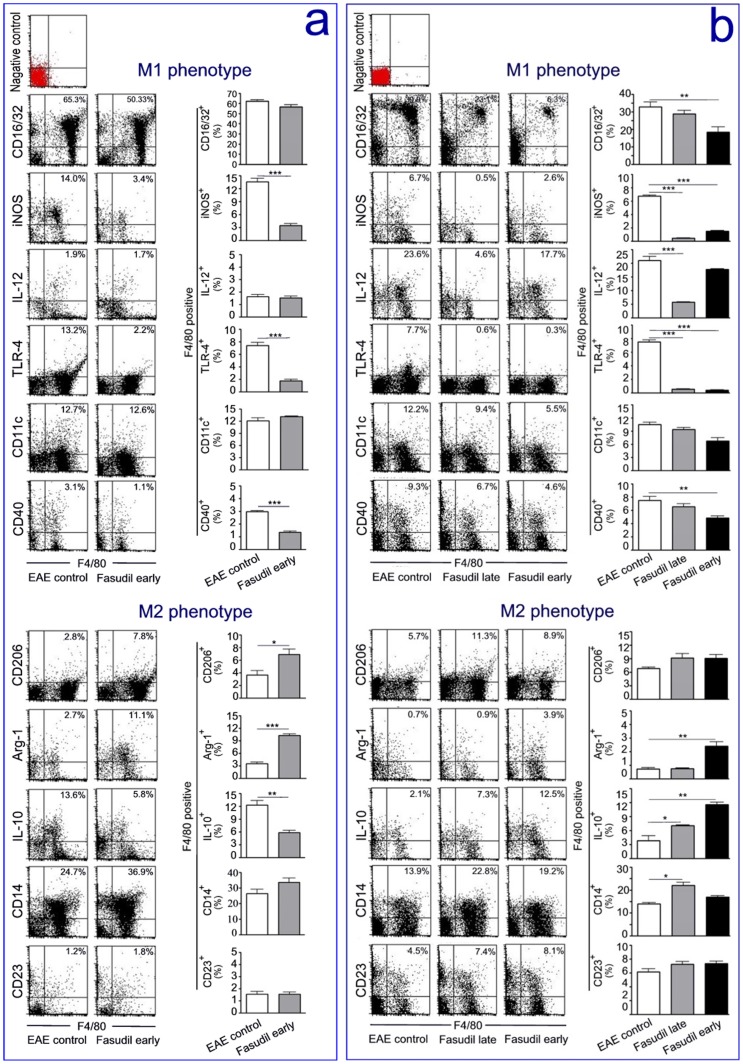
Fasudil shifts M1 to M2 phenotype. Chronic EAE was induced in C57BL/6 mice with MOG_35–55._ Fasudil was injected (i.p.) at 40 mg/kg/d every other day on day 3 and 11 p.i. Mice were sacrificed and splenic MNCs were prepared on day 9 (a) and 21 (b) p.i.. Cells were stained with macrophage marker F4/80 and M1/M2 markers, and the polarization of M1/M2 was analyzed using flow cytometry. Scatterplot, representative dot plots from one of three experiments with similar results. Histogram, quantitative results are analyzed for 5 mice in each group. Results are expressed as the percentage of double positive cells in four-quadrant diagram. *p<0.05, **p<0.01, ***p<0.001.

To further characterize the changes of macrophage polarization, we assessed the M1 markers CD16/32, iNOS, IL-12, TLR-4, CD11c and CD40 as well as the M2 markers CD206, Arg-1, IL-10, CD14 and CD23 on days 9 and 21 p.i. by flow cytometry. On day 9 p.i., phenotypic analysis of macrophage showed that M1 markers iNOS, TLR-4 and CD40 were significantly declined in Fasudil early-treated mice compared with EAE control (Fig. 5a, *p*<0.001, respectively). On the contrary, M2 markers CD206 and Arg-1 were significantly increased in Fasudil early-treated mice compared with EAE control (Fig. 5a, *p*<0.05 and *p*<0.001, respectively).

On day 21 p.i., phenotypic analysis of macrophage showed that three major M1 markers CD16/32, iNOS and IL-12 were significantly declined in Fasudil late- or early-treated mice compared with EAE control (Fig. 5b, *p*<0.01 and *p*<0.001, respectively), especially iNOS. Two additional M1 markers TLR-4 and CD40 were also significantly reduced in Fasudil late- or early-treated mice compared with EAE control (Fig. 5b, *p*<0.01 and *p*<0.001, respectively), especially TLR-4. In contrast, one major M2 marker Arg-1 was significantly increased in Fasudil early-treated mice compared with EAE control (Fig. 5b, *p*<0.01). Two additional M2 markers IL-10 and CD14 were also significantly elevated in Fasudil late- or early-treated mice compared with EAE control (Fig. 5b, *p*<0.05 and *p*<0.01, respectively). The expression of CD11c and CD23 did not differ among the three groups. Our results indicate that administration of Fasudil can shift macrophages from M1 to M2 phenotype in early and late treatment of EAE induction.

**Figure 6:**
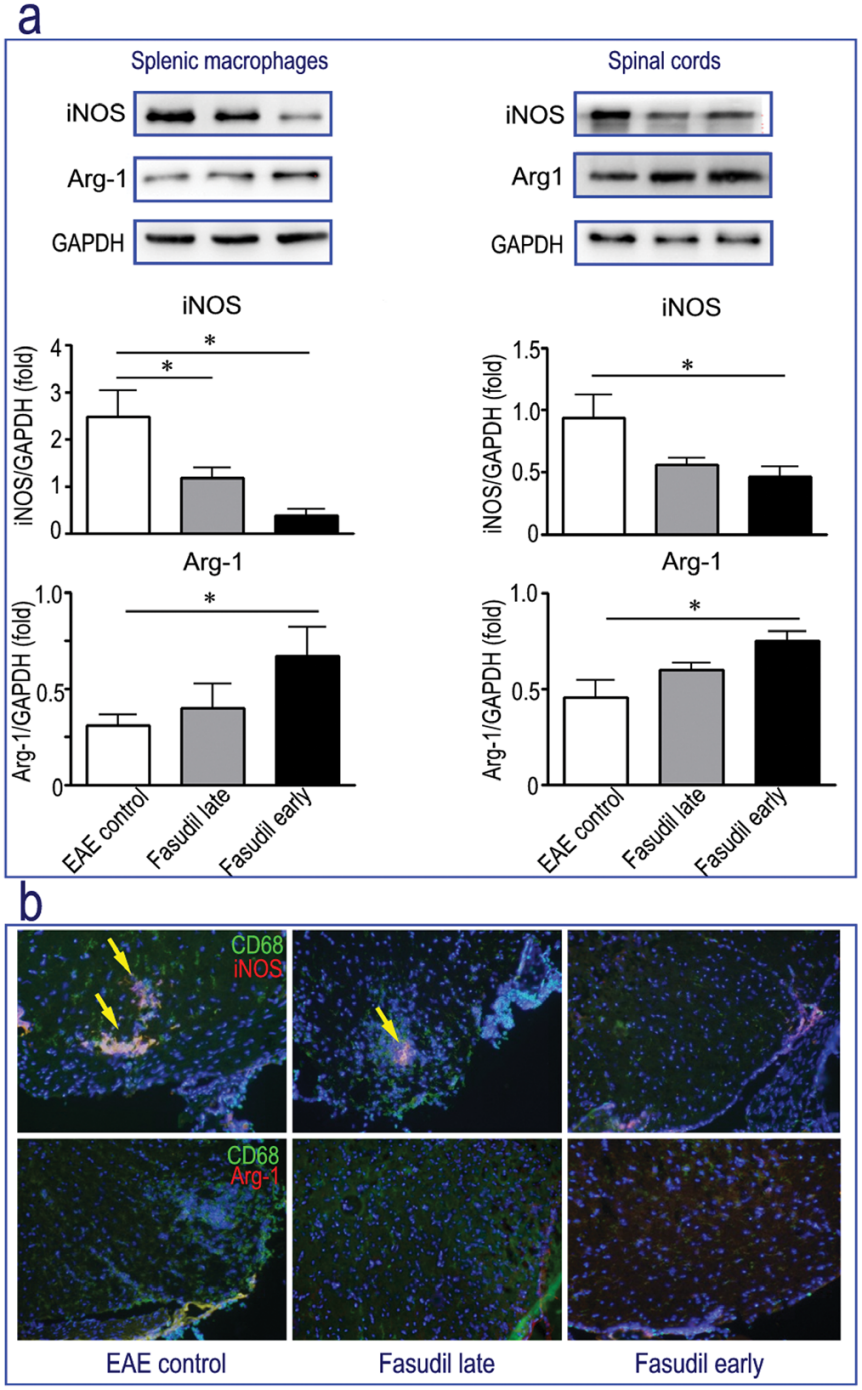
Fasudil enhances the ratio of Arg-1/iNOS. Chronic EAE was induced in C57BL/6 mice with MOG_35–55._ Fasudil was injected (i.p.) at 40 mg/kg/d every other day on days 3 and 11 p.i. On day 21 p.i., mice were sacrificed, and protein was extracted from splenic macrophages and spinal cords. a) Representative bands of Western blot for iNOS and Arg-1 from one of two experiments with similar results. Quantitative analysis of iNOS and Arg-1 expression in splenic macrophages and spinal cords are analyzed for 4–5 mice in each group. Results are expressed as fold change relative to GAPDH as the loading control. *p<0.05, **p<0.01, ***p<0.001. b) The double staining of CD68 (green) and iNOS/Arg-1 (red) by immunohistochemistry. Representative patterns were obtained from one of three experiments with similar results.

Based on literature review, iNOS and Arg-1 are two representatives of M1 and M2 macrophages. We next measured the expression of M1 iNOS and M2 Arg-1 in splenic macrophages and spinal cords by Western blot. As shown in Fig. 6a, Fasudil significantly inhibited the expression of iNOS and enhanced the expression of Arg-1 in both splenic macrophages and spinal cords, especially in Fasudil early-treated mice (*p*<0.05, respectively). To further define these results, we observed the expression of iNOS and Arg-1 on infiltrated CD68^+^ macrophages in spinal cords by double immunohistochemistry. Fig. 6b showed that Fasudil decreased CD68 macrophages that express iNOS (see arrow). In spinal cords of EAE mice, CD68 macrophages expressing Arg-1 are difficult to be detected, revealing that M2 macrophages expressing Arg-1 may be not easy to enter the CNS. Taken together, these results are consistent with the results from flow cytometry as described in Fig. 5.

**Figure 7:**
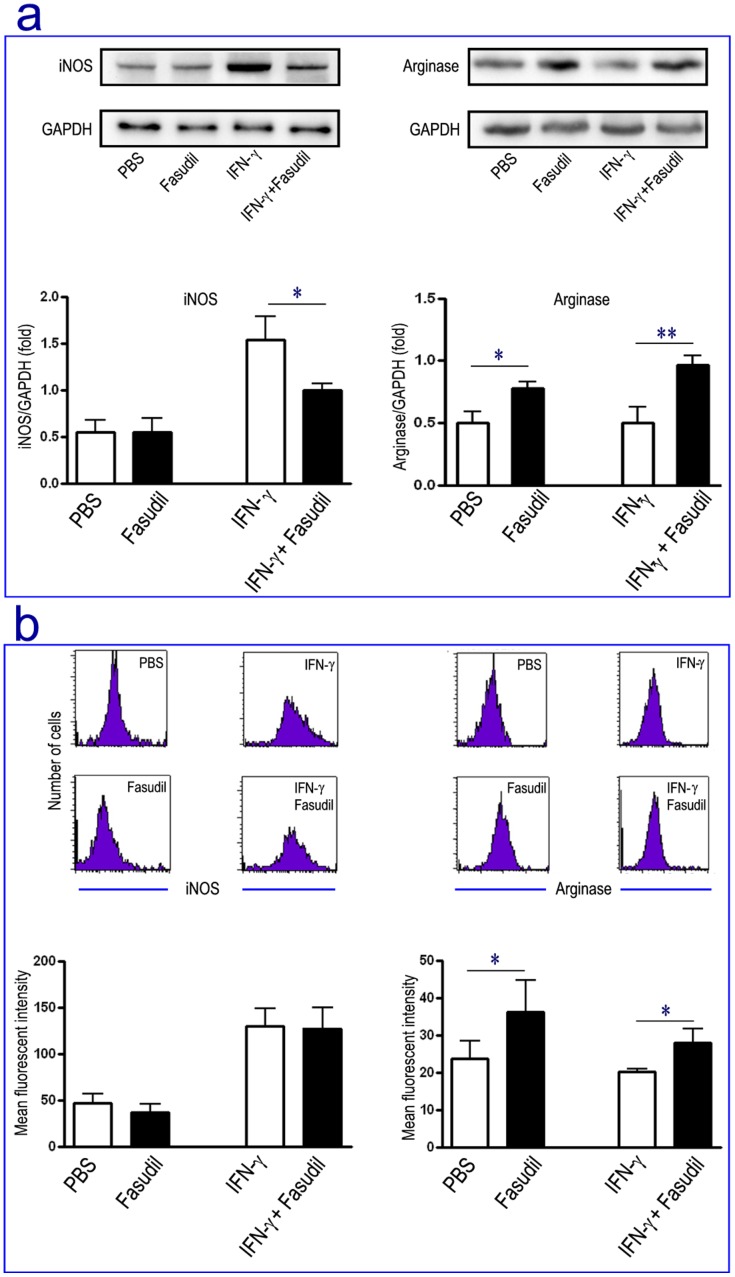
Fasudil directly shifts M1 to M2 phenotype in macrophages. Macrophages from healthy mice were purified and stimulated with IFN-γ. Fasudil was added for 12 h immediately after IFN-γ treatment. The levels of iNOS and Arg-1 were measured by Western blot (a) and flow cytometry (b). The results were analyzed from three independent experiments. *p<0.05, **p<0.01.

Next question is whether Fasudil directly act on macrophages. We established an in vitro experiment to explore the role of Fasudil on macrophage polarization in IFN-γ-stimulated macrophages. The results showed that Fasudil inhibited the level of iNOS and elevated the level of Arg-1 by Western blot (Fig. 7a). Simultaneously, Fasudil increased the expression of Arg-1 in un-stimulated and IFN-γ-stimulated macrophages by flow cytometry (Fig. 7b). Taken together, our results clearly demonstrate that Fasudil shifts M1 to M2 phenotype in early and late treatment of EAE induction, which does not require the participation or auxiliary of other cells.

### Fasudil Suppresses Inflammatory Cytokines and Enhances Anti-inflammatory Cytokine

**Figure 8:**
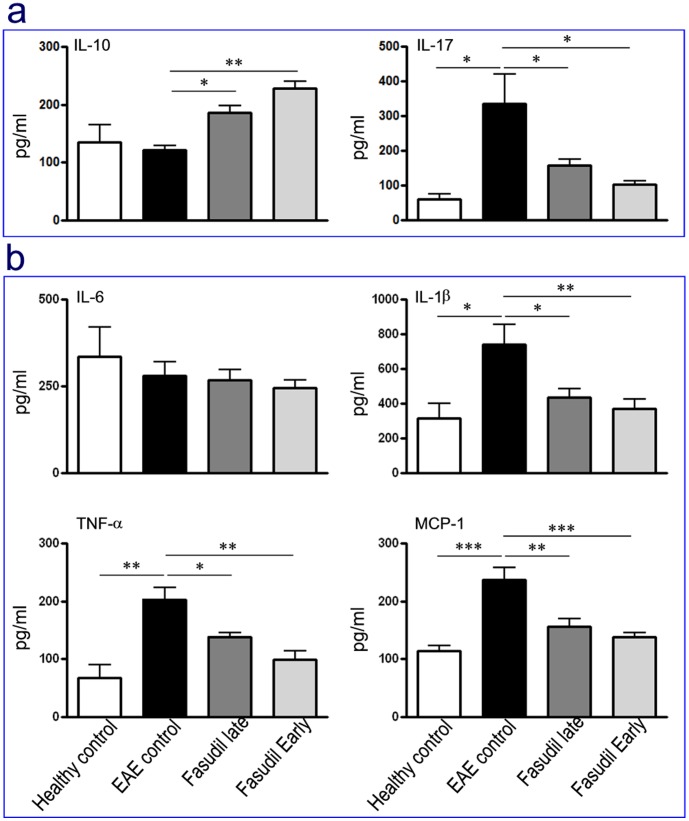
Fasudil inhibits inflammatory molecules. Chronic EAE was induced in C57BL/6 mice with MOG_35–55._ Fasudil was injected (i.p.) at 40 mg/kg/d every other day on days 3 and 11 p.i. On day 21 p.i., spleen was obtained, and splenic MNCs were prepared. Splenic MNCs were incubated for 48 h at 37°C in the presence of MOG_35–55_ (10 µg/ml). Splenic MNCs from healthy mice were measured as control. The supernatants were collected, and the levels of IL-10, IL-17 (a), IL-1β, IL-6, TNF-α and MCP-1 (b) were measured by ELISA kits. The results were represented as pg/ml, and analyzed from 5 mice in each group. *p<0.05, **p<0.01.

Because Fasudil has been shown to shift M1 to M2 phenotype macrophages in spleen, we next measured the levels of inflammatory and anti-inflammatory cytokines such as IL-1β, IL-6, TNF-α, MCP-1, IL-17 and IL-10 by ELISA kits. As expected, treatment of Fasudil significantly inhibited the production of IL-17, IL-1β, TNF-α and MCP-1 in spleen (*p*<0.05 and *p*<0.01 for IL-1β; *p*<0.05 and *p*<0.01 for TNF-α; *p*<0.01 and *p*<0.001 for MCP-1 and *p*<0.01 for IL-17) (Fig. 8a and b). In contrast, the level of IL-10 was elevated in both mice with Fasudil late- and early-treated mice compared with EAE control (*p*<0.05 and *p*<0.01, respectively) (Fig. 8a).

## Discussion

Fasudil, initially characterized as an intracellular calcium antagonist, has been administered to patients with vasospasm after subarachnoid hemorrhage in Japan since 1995. Because of minimal side effect and multi-target effect, Fasudil exhibits more therapeutic potential in several neurological disorders. Previous studies have demonstrated that Fasudil ameliorated severity of EAE partly by decreasing BBB permeability [31] and inflammatory cell infiltration in the CNS [32], [33]. In SJL/J mice induced with PLP_139–151_, Fasudil reduced the proliferation of specific T cells, together with a down-regulation of IL-17 and a decrease of the IFN-γ/IL-4 ratio [32]. In the present study, Fasudil delayed onset of EAE and ameliorated severity of symptoms when administrated in early and late stages of EAE induction, but a better therapeutic effect could be observed at the initial phase of EAE (i.e. on day 3 p.i.), which represents the immunological incipience of the disease.

In the present study, Fasudil reduced inflammatory response and improved demyelination in spinal cords of EAE. Fasudil inhibits the migration of inflammatory cells into the CNS, which may have two explanations: 1) Fasudil improves the permeability of BBB. Previous work from other groups showed that ROCK mediated occludin and claudin-5 phosphorylation resulting in diminished barrier tightness and enhanced monocyte migration across BBB [34]; 2) ROCK plays a key role in cell migration by regulating cytoskeletal dynamics and cell adhesion. It has been reported that ROCK is required for efficient T-cell polarization and migration on endothelial cells as well as transendothelial migration [35].

Infiltrating cells in spinal cord of EAE are mainly composed of T cells and macrophages. Although adoptive transfer studies have established that T cells are both necessary and sufficient for induction of EAE [36], [37], the cellular mechanisms that govern disease progression are still being debated. The dynamic balance between the effector Th1/Th17 and Treg cells controls the development of EAE. Our results expectedly reveal that Fasudil induced CD4^+^IL-10^+^ T cells, and inhibited CD4^+^Th17^+^ T cells compared with EAE control. However, besides T cells, macrophages also play indispensible roles in the development of EAE [38]. Ajami et al. [39] pointed out a three-step model of EAE progression. 1) CD4 T cells are responsible for disease initiation. 2) The mechanisms excluding blood-borne monocytes from the CNS break down. 3) Infiltrating blood-derived monocytes trigger, directly or indirectly, EAE progression. Thus, blood-derived macrophages have been implicated in the development of EAE because of their ability to present antigens, secrete inflammatory cytokines [40] and participate in demyelination by phagocytosis of degraded myelin [41].

The most important result in this study is that Fasudil polarizes M1 into M2 macrophages in EAE mice, as detected *in vivo and in vitro* by using different assays including flow cytometry, Western blot, biochemistry and immunohistochemistry. Based on *a* large amount *of literatures* in the relevant field of macrophage polarization, we selected the markers of M1 polarization, such as CD16/32, iNOS and IL-12 as well as the markers of M2 polarization, such as CD206, Arg-1 and IL-10 [42]–[44]. At the same time, we also selected some other markers of M1 macrophages, such as TLR-4, CD11c and CD40 [42], [45]–[47] as well as the markers of M2 macrophages, such as CD14 and CD23 [42], [48]–[49]. Macrophages are classified into classical M1 macrophages (iNOS positive) and alternatively activated M2 macrophages (Arg-1 positive), which vary according to their induction factors, cytokine production, and phagocytosis type [21], [50]. What factors regulate macrophage polarization? CD4^+^ T-cells play an important role in polarization of macrophages [51]. The polarization of macrophages into M1 or M2 depends on the cytokine milieu in the tissue. Cytokines produced by Th2 T cells would thus polarize the anti-inflammatory M2 macrophages. Fasudil likely regulates the polarization of macrophages, directly or indirectly through Fasudil-mediated CD4^+^IL-10^+^ regulatory T cells or IL-10. However, M2 macrophages exhibit enhanced IL-10 when challenged by LPS [52]. Interestingly, IL-10 itself can also promote M1 to M2 transition [52]. Thus, why Fasudil inhibited the expression of IL-10 in early treatment of EAE induction still needs further investigation.

M2 macrophages are believed to participate in the blockade of inflammatory responses and promotion of tissue repair [53]. The imbalance of M1/M2 macrophages as a key factor of inflammation severity is confirmed in relapsing EAE rat [15], in which treatment with M2-macrophages suppressed EAE. It was reported that IL-33 attenuates the development of EAE possibly through the induction of IL-5 and IL-13 production together with M2 polarization [54]. Administration of in vitro activated M2 monocytes/macrophages suppressed ongoing severe EAE and increased immunomodulatory expression pattern in lesions, demonstrating the concept that drugs favoring M2 differentiation are promising therapeutic approaches [15]. Thus, we propose that Th2 cytokines may contribute to the polarization of M2 macrophages, while M2 macrophages in turn influence the balance of Th1/Th17 and regulatory T cells, constituting a micro-environment that contributes to myelin repair and inflammatory suppression.

In conclusion, Fasudil delays onset, and ameliorates severity in different phases of EAE induction, accompanied by improvement of demyelination and inhibition of inflammation. Interestingly, Fasudil shifts M1 to M2 macrophage phenotype, producing less inflammatory cytokines. Our findings provide a rationale for a therapeutic strategy, which might have potentially fewer side effects than existing therapies. However, the exact function of Fasudil in treatment of EAE still merits further investigation.
